# Internalized stigma among patients with mood disorders in Ethiopia: a cross-sectional facility-based study

**DOI:** 10.1186/s13033-020-00365-8

**Published:** 2020-05-06

**Authors:** Elias Tesfaye, Benyam Worku, Eshetu Girma, Liyew Agenagnew

**Affiliations:** 1 Department of Psychiatry, Faculty of Medical Sciences, Institute of Health, Jimma University, Jimma, Ethiopia; 2 Department of Psychiatry, School of Medicine, College of Health Sciences, Addis Ababa University, Addis Ababa, Ethiopia; 3 Department of Preventive Medicine, School of Public Health, Addis Ababa University, Addis Ababa, Ethiopia

**Keywords:** Internalized stigma, Mood disorder, Disability, Ethiopia

## Abstract

**Background:**

Information on the degree of internalized stigma experienced by patients with mood disorders in Ethiopia is limited. This study attempted to assess the levels of internalized stigma and factors associated with it in patients with mood disorders who were on follow-up as an outpatient in a Psychiatry clinic at Saint Paul’s Hospital, Addis Ababa, Ethiopia.

**Methods:**

A facility-based cross-sectional study employed, and a consecutive sampling technique was used to get study participants (235 cases with mood disorders). Internalized stigma of mental illness scale used to assess stigma of study subjects. The collected data were cleaned, checked for completeness, coded and entered into Epi-data version 3.1 data entry software and exported to SPSS version 20 statistical software for analysis. Univariate linear regression analysis was done to see the association between dependent and independent variables at P-value < 0.25 and multivariate linear regression analysis was done to identify predictor variables at P-value < 0.05.

**Results:**

Nearly one-third (31.5%) of the patients had moderate or high levels of internalized stigma, and more than half (54.9%) of the respondents had moderate or high stigma resistance and self-esteem score of (67.2%). About a quarter (27.7%) had moderate to high levels of discrimination experience and a similar proportion (26.4%) had moderate to severe or extreme disability. Females had significantly higher internalized stigma (std. β = .169 with P < 0.01) than men. Adherence to medication was significantly correlated with lower internalized stigma (std. β = − .212 with P < 0.01).

**Conclusions:**

These findings suggested that moderate to high internalized stigma occurred among approximately 1 in 3 people with a mood disorder in the urban city of Ethiopia. So, working on adherence to medication, self-esteem of patients and psycho-education about stigma is crucial to reducing the internalized stigma of people with a mood disorder and special attention should give to female patients.

## Background

Stigma defined by the World Health Organization as ‘a mark of shame, disgrace, or disapproval that results in an individual being rejected, discriminated against and excluded from participating in many different areas of society’ [1].

Mainly there are two main types of stigma social stigma and internalized or self-stigma.

Social stigma, also known as public or enacted stigma, exists at the group level and a phenomenon of social groups endorsing stereotypes about and acting against a stigmatized group [2, 3]. Whereas internalized stigma or self-stigma exists at the individual level and can be described as a transformative process wherein a person offers up his or her previously held or favored identities as a figure, employee, and partner to adopt a stigmatized, anticipate social rejection, and believe they are devalued members of society [4–7].

Internalized stigma can found in patients with all type of mental illness, but its severity and level of presence is not the same to all type of mental illness [8]. Studies indicated that around 80 to 90% of people with mental illness experience stigma and discrimination in many countries [9].

A study performed in Singapore among psychiatric outpatients showed, 43.6% of participants experienced moderate to high internalized stigma [10], and a study from China indicated 38.3% Hong Kong and 49.5% Guangzhou participants had self-stigma [11].

The prevalence of internalized stigma in Dilla, Ethiopia was 32.1% among people living with mental illness [12], whereas the study performed in Jimma, found the overall self-stigma mean score of 2.32 with SD ± 0.30, in patients with severe mental illness, and among the general respondents, 25.12% of them confirmed 2.5 and above self-stigma score [13].

Patients with schizophrenia had higher mean score of internalized stigma, than those with bipolar disorders as the finding from rural China [14], and Turkey [15], but opposite to this finding from London showed patients with mood disorders experienced more stigma than patients with psychotic disorders [16].

Internalized stigma has plenty of effects on patients with mental illness, especially on the prognosis of illness [6, 17], on quality of life, on self-esteem [18, 19], and their functioning [20, 21]. Also can make patients not to seek treatment for a mental illness, often resulting in delayed treatment-seeking [7, 22] and associated with an increase in symptom severity [23].

Low self-esteem, hope, and well-being, general functioning, age, sex, income, unemployment, education, duration of illness, adherence, having depressive episode, duration of illness, poor social support, presence of medication side effect, recovery, severity of symptoms, insight, family history of mental illness and substance use were factors associated with internalized stigma [10, 12, 24–32].

However previous studies focused on patients with schizophrenia [33, 34], despite both major depression and bipolar disorders are 6th and second cause of disability worldwide [35], and highly correlated to the severity and the impact of stigma [8, 36], less attention given to patients with bipolar disorder and depression, especially in the Ethiopian context. So this study focuses on patients with a diagnosis of bipolar disorder or/and depression aimed to determine the level of internalized stigma and associated factors among patients attending psychiatry outpatient department at St. Paul’s medical hospital, Addis Ababa, Ethiopia, and it will be important for patients and professionals to prevent and manage stigma after identifying the possible factors for it.

## Methods

### Study setting

The study conducted at St. Paul’s hospital, Addis Ababa, Ethiopia. It is a referral hospital in the capital city of Ethiopia. The Hospital built-in 1969. It has 392 beds, with an annual average of 250,000 patients and a catchment population of over 5 million.

It has 13 types of clinical services. Patients with mental illness treated by psychiatrists and psychiatry residents under the supervision of senior Psychiatrists. The hospital has no inpatient services for mentally ill patients except the rehabilitation services for those with a problem of substance use disorder with five beds only. On average, 900 patients with mental illness have been seen per month. Around 11,000 new and old patients with mental illness had received treatment per year.

### Study design

A facility-based cross-sectional study employed.

### Sample size determination and selection of participants

The sample size determined by using a single population proportion formula with the assumptions of 95% confidence interval, Prevalence = 21.7% found from study in Europe [37], with a margin of error of 5%; the required sample size would be 261, and we collected 90% of the expected sample size = 235 in the study period from June to August 2014.

After reviewing their earlier chart record we identified all bipolar disorder and depressed patients. A third-year psychiatry resident confirmed their diagnoses using DSM-IV criteria of bipolar disorder and major depression for those disorders to check its consistency with the earlier diagnosis. If there was a discrepancy between the two diagnoses, we accepted a consultant psychiatrist diagnosis.

#### Inclusion and exclusion criteria

Patients who have a diagnosis of bipolar disorder or depression with having more than one follow-up and aged 18 years and above were included in the study whereas patients who were acutely ill and needing emergency treatment, having comorbid physical illness and who came for the first visit excluded from the study.

### Data collection procedure and instruments

Face to face interviews and document reviews used to collect the data for this study. First-year and second-year psychiatry residents involved in data collection after they took 3 days of training then after permission obtained from respondents, the interview was administered and took approximately 30 to 45 min to complete. The interviews carried out in private rooms, in their order of visit. If they didn’t fulfill the inclusion criteria the next samples recruited consecutively.

Each data collector reviewed the card and recorded the card number of respondents who had completed the questionnaire and share to all data collectors to avoid redundancy of the participants. The principal investigator and the supervisors checked completeness of the questioners and feedback was given to data collectors on a daily base. The only challenge we faced was 26 participants didn’t complete questionnaires which we excluded from the analysis.

### Instruments

#### (1) The internalized stigma of mental illness (ISMI)

A 29-item scale was used to assess the internalized stigma. It has 5 subscales: alienation, stereotype endorsement, perceived discrimination, social withdrawal, and stigma resistance. Alienation is the personal experience of being less than a full member of society [34]. The stereotype endorsement is the degree to which the client’s opinion agrees with common stereotypes about patients with a mental illness [13]. The discrimination experience measures the way the client tends to be treated by others [38]. The social withdrawal measures the tendency of the individual to be excluded from social activities due to mental illness [13]. The stigma resistance subscale is a respondents’ ability to defy stigma [38]. In contrast to the above four subscales, a higher score on the stigma resistance subscale indicates a lower level of stigma resistance [38].

In a study done in Iran, the internalized stigma of mental illness scale subscales had the following reliability values: alienation = 0.84, stereotype endorsement = 0.71, discrimination experience = 0.87, social withdrawal = 0.85 and stigma resistance = 0.63 [37]. And also, the scale had a strong internal consistency (α = 0.90) and test–retest reliability (r = 0.92) have been reported for the internalized stigma of mental illness scale [19].

In the previous study in Ethiopia, the internalized stigma of mental illness scale subscales had reliability values of alienation = 0.84, stereotype endorsement = 0.73, discrimination experience = 0.79, social withdrawal = 0.77 and stigma resistance = 0.65 [13].

Recent studies have shown that the ‘stigma resistance’ subscale is theoretically different from the other subscales [39, 40]. For this reason, stigma resistance considered as a separate construct to the internalized stigma of mental illness throughout this paper. Stigma resistance subscale reversely coded (4 = strongly disagree to 1 = strongly agree). Participants responded to each internalized stigma of mental illness scale question by selecting: 1 for strongly disagree; 2 for disagree; 3 for agree; 4 for strongly agree. The overall internalized stigma of mental illness score refers to the summed average of the other four internalized stigma of mental illness scale subscales excluding the stigma resistance subscale. A higher score shows higher internalized stigma.

#### (2) The Rosenberg self-esteem scale

The Rosenberg self-esteem scale used to measure the level of self-esteem. The scale has 10 items. Some items are reverse coded (1 = strongly disagree to 4 = strongly agree). The respondents provided 1 for strongly disagree; 2 for disagree; 3 for agree; 4 for strongly agree. Higher scores show higher self-esteem. Although not formally validated, this version used in a study conducted in Ethiopia [13], which suggests that it was appropriate to use as a stand-alone measure.

#### (3) WHO Disability Assessment Schedule version 2 (the 12 items)

The 12-item interviewer-administered Disability Assessment Schedule version 2 (WHODAS-II), developed by the World Health Organization used to explain the level of disability associated with depression and bipolar disorders. WHODAS assesses the level of disability and the number of days lost from work in the previous 30 days. The instrument is considered cross-culturally applicable [41, 42]. The Amharic version of the 36-item version has been previously used in Ethiopia [43].

#### (4) Previous suicidal attempt

A question on suicidal attempt was presented to the participants by asking “Have you ever felt so desperate that you even attempted to hurt yourself or end your life?” [44]. this question was similar to a question used in asking about the suicide attempt in the widely used structured composite international diagnostic interview instrument of the World Health Organization [44].

#### (5) Medication adherence

The medication adherence question assessed about a history of non-adherence with psychotropic medications and whether the non-compliance behavior linked to stigma. It has inquired “Have you ever stopped taking medication?” and “did your personal experience of stigma contribute to your decision to discontinue medication?”

#### (6) Current clinical status

The patient’s clinical status measured by using diagnostic statistical manual fourth edition diagnostic criteria of bipolar disorder and depression, and saying fully improved (if they didn’t meet the criteria), partially improved (if they have a clinical sign and symptoms), not improved (if they have fulfilled the criteria), and to say a patient had a relapse history the patient should have a history exacerbation of symptoms or more than one admission history which was measured by reviewing the card and interviewing patients.

#### (7) Structured questioners

Structured questioners were used to assess Sociodemographic variables (sex, age, educational level, marital status, employment status, and monthly income) and clinical variables (working diagnosis, (age at the onset of illness, the total duration of illness, duration of treatment delay and duration of treatment which we treat those variables as a continuous and explained by mean and SD)).

### Data processing and analysis

The collected data were cleaned, checked for completeness, coded and entered into Epi-data version 3.1 software and exported to SPSS version 20 statistical software for analysis. Descriptive statistics used to summarize the results.

Simple linear regression used to identify variables that are a candidate for multiple linear regressions at P-value < 0.25. A multivariate linear regression model was done at P-value < 0.05 using variables that had significant statistical associations with the overall internalized stigma of mental illness scores during Univariate analysis. Finally, predictor variables were declared at P-value < 0.05 and the obtained results presented using narration and tables.

All assumption of linear regression analysis was checked and the data was found to meet the assumptions such as normality was checked by using normal histogram curve and Kolmogorov–Smirnov Test, linearity was checked by using (Quantile–Quantile) QQ plot and histogram, no-outlier was found by using outlier test, multicollinearity was checked by using variables inflation factor and all variables had VIF < 2, homoscedasticity checked by using Levene’s test and all variables found to have P > 0.05. Independent observation checked by Durbin Watson’s value and the value of this finding was 2.

### Ethical consideration

Ethical clearance was obtained from the ethical review board of Addis Ababa University, besides written informed consent was sought from each participant.

## Results

### Sociodemographic characteristics of study participants

There were 235 samples take part in the study with a 90.4% response rate. Most of them were females (60.4%). The mean age was 37.94 with (SD ± 13.2) years. More than one-third of the participants 92 (39.1%) were married and 104 (44.3%) were single in marital status. Most participants 218 (92.8%) were educated. More than one-fourth of them (27.7%) were unemployed and nearly half (46.4%) were either government or private employees (Table 1). Around half of them (42.6%) had no income and the mean monthly income of the participants in USD was about 59.65 (SD ± 85.7).

**Table 1:** Background characteristics of respondents at St Paul’s hospital, Addis Ababa, Ethiopia, 2014

Characteristics	Frequency	Percent
Sex		
Male	93	39.6
Female	142	60.4
Marital status		
Single	104	44.3
Married	92	39.1
Divorce or separated	19	8.1
Widowed	20	8.5
Job-status		
Unemployed	65	27.7
Daily laborer	2	.9
Housewife	25	10.6
Student	24	10.2
Farmer	5	2.1
Retired	5	2.1
Government employee	53	22.6
Private employee	56	23.8
Educational level		
Illiterate	17	7.2
Education from grade 1 up to grade 8	54	23
Education from grade 9 up to grade 12 or (10 + 2)	71	30.2
College or university	93	39.6

### Clinical characteristics of study participants

Out of all participants, more than one-third (37.4%) had bipolar disorder and nearly two-thirds (62.6%) had a depression diagnosis. The Mean age onset of the illness was 27.89 (SD ± 11.2). The mean total duration of the illness was 10 years (SD ± 9.4). The mean duration of treatment was 7.45 year with (SD = 8.1), and the Mean duration of treatment delay was 2.6 year with SD ± 4.8. Nearly, nine out of ten (86.4%) claimed to be either partially or fully improved. Over one-third of them (35.7%) had a history of suicidal attempts. And also, half of them (48.5%) had a history of treatment non-adherence, from those participants who were non-adherent to medication, almost one-third (29.8%) claimed that stigma played a role in their non-adherence (Table 2).

**Table 2:** Clinical characteristics of respondents at St Paul’s hospital, Addis Ababa, Ethiopia, 2014

Clinical characteristics	Frequency	Percent
Diagnosis		
Bipolar	88	37.4
Depression	147	62.6
Level of improvement to treatment		
Fully improved	112	47.7
Partially improved	91	38.7
No change	9	3.8
Relapse	23	9.8
Suicidal attempt		
Yes	84	35.7
No	151	64.3
Medication non-adherence		
Yes	114	48.5
No	121	51.5
Contribution of stigma to non-adherence (N = 114)^a^		
Yes	34	29.8
No	80	70.2

^a^Those without a history of non-adherence (N = 121) excluded

### Scoring and scaling structures

The internal consistency for the 24-item internalized stigma of mental illness scale (excluding stigma resistance) was α = 0.95. The stigma resistance subscale had an internal consistency of α = 0.87. The other 4 subscales had the following values: alienation (α = 0.90), stereotype endorsement (α = 0.79), discrimination experience (α = 0.88) and social withdrawal (α = 0.95). The Rosenberg self-esteem questioner had an internal consistency of α = 0.80. And also, the WHODAS II (the 12 items) had an internal consistency α = 0.96 (Table 3).

**Table 3:** Summary of Cronbach’s Alpha of the scales used in comparison with other studies

Subscales	Finding from this study	Girma et al. [13]	Brohan et al. [37]	Ghanean [38]
Alienation	0.90	0.84	0.83	0.84
Stereotype	0.79	0.73	0.81	0.71
Discrimination	0.88	0.79	0.83	0.87
Withdrawal	0.95	0.77	0.85	0.85
Overall 24 ISMI scales	0.95	0.89	0.94	–
Stigma resistance scale	0.87	0.65	0.59	0.63
Self-esteem scale	0.80			
WHODAS II (the 12 items)	0.96			

### Internalized stigma of mental illness scale

Overall internalized stigma of mental illness (excludes Stigma resistance): the overall mean of the 24 internalized stigma of mental illness scale was 2.2 with (SD ± 0.63). Using mean score categories similar to the European study (< 2 minimal stigma, 2–2.5 low stigma, 2.5–3 moderate stigma, 3+ high stigma); Among the total respondents, almost one-third (31.5%) of the participant with mood disorder had moderate to high level of internalized stigma of mental illness considering the mean cutoff point ≥ 2.5. Overall, 80.4% of the participant had responded by saying agree or strongly agree with at least one item in the internalized stigma of mental illness scale.

The mean score of alienation was 2.56 with SD ± 0.82. More than half of them (54.5%) had moderate to high levels of alienation. The mean score of stereotype was 2.02 with SD ± 0.59. Around one-fifth of them (21.7%) had moderate to high levels of stereotype endorsement. The mean of discrimination was 2.13 with SD ± 0.77. More than a quarter (27.7%) of the participants had moderate to high levels of discrimination experience. The mean of social withdrawal was 2.11 with SD ± 0.74. (26.4%) had moderate to a high level of social withdrawal. The mean of stigma resistance was 2.6 with SD ± 0.78. More than half (54.9%) had moderate to the high level of stigma resistance score as shown in (Tables 4 and 5).

**Table 4:** Comparison of mean scores and standard deviation of stigma domain with the study in Europe

Stigma domain	Mean and standard deviation			
	Ethiopia		Europe	
	Mean	SD	Mean	SD
Overall ISMI (excludes stigma resistance)	2.2	0.63	1.79	0.87
Alienation	2.56	0.82	2.22	1.09
Stereotype endorsement	2.02	0.59	1.59	0.78
Discrimination experience	2.13	0.77	1.91	0.96
Social withdrawal	2.11	0.74	1.98	1.00
Stigma resistance	2.6	0.78	2.81	0.98

**Table 5:** Prevalence of internalized stigma and WHODAS II scales of respondents in comparison with a study in Europe

Scale	Prevalence of moderate to high levels in our study (%)	Results from a study in Europe [37] (%)
Overall self-stigma (excludes Stigma resistance)	31.5	21.7
Alienation	54.5	39.4
Stereotype endorsement	21.7	12.5
Discrimination experience	27.7	21.7
Social withdrawal	26.4	28.7
Stigma resistance	54.9	59.7
Self-esteem	67.2	–
WHODAS II (the 12 items)	26.4	–

*Self-esteem and WHODAS* The mean of self-esteem was 2.6 with SD ± 0.44. (67.2%) had moderate to high self-esteem considering the mean cutoff point to be ≥ 2.5. The mean of WHODAS II (the 12 items) was 2.13 with SD ± 1.24. One-fourth of them (26.4%) had moderate to severe or extreme disability WHODAS II (the 12 items) score considering the mean cutoff point ≥ 3 (Table 5).

The mean number of days of disability in 30 days was 10.7 with SD ± 12.05. The mean number of days of total difficulty in 30 days was 1.93 with SD ± 5.26. The mean number of days of partial difficulty in 30 days was 8.17 with SD ± 10.95.

### Factors associated with internalized stigma of mental illness

A bivariate linear regression done after appropriate variables converted to a dummy variable. Being married (std. β = − .204) compared to being divorced or separated (std. β = − .177); increment in age (std. β = − .200); increment in income (std. β = − .236); increment in the duration of treatment (std. β = − .177); being fully improved when compared with being partially improved (std. β = − .318), no change (std. β = − .192) or relapse (std. β = − .338); good adherence history to medication (std. β = − .212) when compared with non-adherence; and increment in self-esteem score (std. β = − .635) associated with decreased internalized stigma scores.

Being female (std. β = .169) when compared with being male; having positive history of suicidal attempt (std. β = .140) when compared with those with no history of suicidal attempt; high stigma resistance score (std. β = .719); high level of WHODAS II (the 12 items) disability score (std. β = .513); large number of days of difficulty to function (std. β = .430); large number of days of total difficulty to function (std. β = .325); and large number of days of partial difficulty to function (std. β = .316) was significantly associated with increased internalized stigma scores (Table 6).

**Table 6:** Univariate and multivariate linear regression analysis results in respondents at St Paul’s hospital, Addis Ababa, Ethiopia 2014

Independent variables	Bivariate results		Multivariate results	
	Standardized beta coefficient	P-value	Standardized beta coefficient	P-value
Single (reference married)	.204	.004**	.046	.420
Divorce or separated (reference married)	.177	.009**	.072	.123
widowed (reference married)	.023	.730	.003	.946
Male (reference female)	− .169	.009**	.026	.568
Age of the patient	− .200	.002**	.098	.441
Monthly income	− .236	.000**	− .020	.681
Duration of treatment	− .177	.006**	.011	.903
Partially improved (reference fully improved)	.318	.000**	.049	.333
No change (reference fully improved)	.192	.002**	.004	.932
Relapse (reference fully improved)	.338	.000**	.016	.755
No non adherent (reference yes non adherent)	− .212	.001**	− .029	.529
Mean of self− esteem	− .635	.000**	− .274	.000**
Mean of stigma resistance	.719	.000**	.451	.000**
Mean of WHODAS II (the 12 item)	.513	.000**	.114	.078
H1(the overall disability days in the past 30 days)	.430	.000**	− .184	.294
H2 (the days of total difficulty to function in the past 30 days	.325	.000**	.144	.113
H3 (the days of partial difficulty to function in the past 30 days)	.316	.000**	.202	.214
Age at the onset of illness	− .143	.028*	− .229	.026*
No suicide history (reference yes)	− .140	.031*	.025	.581

** Those with P-value less than 0.01

* Those with P-value less than 0.05

No statically significant association was found between the mean of overall internalized stigma with job status, educational level, treatment delay, duration of illness, the client response to the question “does stigma contribute to nonadherence” and with diagnosis (being as the case of bipolar disorder or depression). Even though, there was no statistically significant difference between the mean score of the overall internalized stigma of mental illness of bipolar and depression; there was a statistically significant mean difference in the subscales. In alienation and stereotype, subscales Patients with major depressive disorder, have a higher level of internalized stigma than patients with bipolar disorder but not in discrimination and withdrawal subscales.

Level of self-esteem, level of stigma resistance, and age at the onset of illness were independent predictors of internalized stigma in the multivariate linear regression analysis with P-value < 0.05.

As the level of self-esteem increased the level of self-stigma decreased with (std. β = − .274 with P < 0.01); as the level of stigma resistance score increased the level of self-stigma increased with (std. β = .451 with P < 0.01); and also as age at the onset of illness increased the level of self-stigma decreased with (std. β = − .229 with P < 0.05).

The linear regression model explained 59.9% of the variance on internalized stigma with a P-value < 0.05.

## Discussion

This study showed a high burden of internalized stigma among patients with mood disorders attending a psychiatric outpatient department in the urban city of Ethiopia. Almost one-third (31.5%) of participants with a mood disorder in this study reported moderate to high levels of internalized stigma and it became corresponding to the study conducted in Dilla university referral hospital, Ethiopia (32.1%) had a high level of self-stigma even though maximum patients diagnoses had been psychosis [12].

Moreover, it’s far much like the finding from North India about, 28% of bipolar disorder patients stated moderate to high level of self-stigma [24]. This is also similar to the study performed in the US branch of Veterans Affairs medical center; about 28% of psychiatric outpatients pronounced a high level of internalized stigma [18]. However it was lower than the look at in Tehran, Iran 40% of respondents had an average scoring of 2.5 or above [38]. The distinction might be due to patients with severe mental illness included and cultural, and Sociodemographic deference between the participants.

This finding became higher than the record from Iran (26.7%) and, Jimma university hospital (25.12%) which conducted among Bipolar-I disorder and other mental illness respectively [13, 45]. This result variation is probably as it is focused on bipolar one disorder patients and patients with mental illness but our study was on patients with bipolar disorder and depression and Sociodemographic distinction. And it became additionally higher than from North-Eastern Nigeria finding 22.5% of patients had a high internalized stigma [28]. The distinction might be because of sufferers with schizophrenia were included. However, the study was lower than the finding from Laagos, Nigeria (21.6%) [30], this difference might be because of other groups of mental illness included.

On this study, the overall mean score of internalized stigma of mental illness across in all 4 domain was between 2.0 and 2.6 in our look at, this is relatively higher than the mean score in European research [37]; indicating a clean massive distinction in the experience of internalized stigma of mental illness among low and high-income nations.

Females had higher self-stigma than Males which is similar to a study in Jimma university hospital, Ethiopia [13]. This might be explained by Females being exposed to a more blaming explanation of mental illness and social disadvantages. But it is in contrast to the study from China males’ demonstrated highest internalized stigma than females [27]. In other findings from China and Maryland shows no significant differences between Male and Female patients (14.30).

As age increased the stigma mean score was decreased, which is similar to a study conducted in European countries and Ethiopia [34, 37, 46]; however, this finding became inconsistent with the study from China and Maryland, no variations in mean scores of internalized stigma of mental illness among adults (18–64 years) and the elderly (≥ 65 years), [14, 29].

Having high-income status decreases the stigma score, which is similar to the finding from china [5]. The reasons may be related to patients who have a lower level of income may easily, have low self-esteem and a higher score of internalized stigma.

Being married decrease the stigma score as compared to being divorced or separated, which is in line with the finding from China [14]. The feasible reason may be associated with the fact that those being unmarried and divorced should face extra discrimination from family and local groups.

Having a good adherence to the medication decreases the stigma score as compared to not having good adherence, which is in line with the finding from Dilla university referral hospital, Ethiopia [12]. The possible reason might be taking medications are directly associated with disclosure of their health status, and may lead to decrease stigma.

Having full improvement decrease the stigma score as compared to having partial improvement this might be because of patients who have improvement or no sign and symptoms and have good functional status, they have insight about the illness and adhere to their medication which ends up a low self-stigma score.

Having no history of suicidal attempt to decrease the stigma score as compared to having a history of suicidal attempt in this study but finding from Poland indicates suicidal attempt not associated with internalized stigma [26]. This difference might be due to our study is specific to mood disorders which have a high risk of suicide.

No statistical difference was observed in the overall stigma score concerning diagnosis in this study in contrast to the finding from china [14]. But in alienation and stereotype subscales Patients with major depressive disorder, have a higher level of internalized stigma than patients with bipolar disorder but not in discrimination and withdrawal subscales.

In this study, job-status has no association with the internalized stigma score which is in line with the finding from Poland [26].

The finding from this study showed as the educational level of patients has no statistical association in stigma score and this is opposite to the finding from china and Poland [27]. A possible explanation might be better education makes humans’ having a greater awareness of their disease, which can be related to their belief and mindset which ends up with a low stigma score.

Treatment delay has no associations with internalized stigma score in this study but it was identified as a significant forecaster of stigma in both Ethiopian and European studies [13, 37]. Similar to study in Europe and Ethiopia [13, 34, 37], this study showed high feelings of alienation but less agreement with common stereotype endorsement about people with a mood disorder. But the opposite relationship was found between self-esteem and the overall internalized stigma of mental illness score and stigma resistance score.

Finally, this study demonstrated a preliminary significant association between Internalized stigma of mental illness and days of total or partial difficulty to function and level of disability (WHODAS II the 12 items) score; suggesting for further study to assess the contribution of internalized stigma on the level of disability and vice versa.

The expected limitation of the study may start from as this is a cross-sectional study there is the difficulty of determining cause-effect relation or time temporal relations between variables.

Finally, while interpreting the findings of this study, it is essential to take into account the fact that it had selection bias, because we didn’t use a random selection of participants and also, being facility base study will affect its generalizability. Lastly not using a standard tool to measure the clinical status of the patients was one of the limitations of this study.

## Conclusions

The findings of this study suggested that internalized stigma was a major problem among persons with a mood disorder in the study area. Approximately one-third of patients with a mood disorder at outpatient follow-up in the urban city of Ethiopia, experience moderate to high levels of internalized stigma.

Internalized stigma had the potential to affect adherence to medication and was more likely to affect the recovery process and productivity. So, we recommend working on enhancing the self-esteem of patients and focused on medication compliance and empowerment interventions with a stronger emphasis on female patients. Therefore, tackling stigma should be a priority in mental health advocacy in low-income countries.

Better if a strategy to minimize stigma be integrated into initiatives to scale up mental health services in low-income countries. Finally, further research is needed to examine the impact of interventions targeted towards people with a bipolar disorder or depression on reducing the Internalized Stigma.

## Data Availability

The datasets used/or analyzed during the current study is available from the corresponding author on reasonable request.

## Abbreviations

DSM-IV: The diagnostic statistical manual fourth edition
ISMI: Internalized Stigma of Mental Illness
WHODAS: World health organization Disability Assessment Schedule
